# Improvement of Spatial Memory and Cognitive Function in Mice via the Intervention of Milk Fat Globule Membrane

**DOI:** 10.3390/nu15030534

**Published:** 2023-01-19

**Authors:** Yongjie Zhou, Xiaoxiao Zou, Ruifang Feng, Xin Zhan, Hui Hong, Yongkang Luo, Yuqing Tan

**Affiliations:** 1 Beijing Laboratory for Food Quality and Safety, College of Food Science and Nutritional Engineering, China Agricultural University, Beijing 100083, China; 2 Department of Product and Development, Heibei Dongkang Dairy Co., Ltd., Shijiazhuang 052165, China

**Keywords:** milk fat globule membrane, memory and cognition, proteomics, DEPs

## Abstract

With the improvement of living standards, dietary interventions have become an appropriate approach to enhance memory and cognitive performance. The present study investigated the potential mechanisms of spatial memory and cognitive function improvement with the milk fat globule membrane (MFGM) intervention in mice. The Morris water maze experiment revealed that the trajectories of mice in group M were more disordered. Also, the immunohistochemical results demonstrated a significantly higher number of neurons in group M compared with group C, especially in the hippocampal dentate gyrus (DG) area. It is suggested that MFGM enhanced mice’s spatial memory and cognition from macroscopic behavior and microscopic cytology, respectively. Meanwhile, 47 differentially expressed proteins (DEPs) were identified, including 20 upregulated and 27 downregulated proteins. Upregulated (Sorbs 2, Rab 39, and Cacna 1e) and downregulated (Hp and Lrg 1) DEPs may improve spatial memory and cognition in mice by promoting synapse formation and increasing neurotransmitter receptors. KEGG enrichment analysis of the DEPs identified seven signaling pathways that were significantly enriched (*p* < 0.05). One of these pathways was neuroactive ligand-receptor interactions, which are strongly associated with improved spatial memory and cognitive performance. These findings give some new insights and references to the potential mechanisms of spatial memory and cognitive enhancement by MFGM.

## 1. Introduction

Stressful events are ubiquitous in our surroundings and are valid for all of us. Stress can negatively affect learning and memory skills, causing alterations such as memory loss and reduced learning ability [1,2]. It is worth mentioning that stress-induced alterations in learning and memory are also thought to contribute to stress-related psychiatric disorders such as major depression or post-traumatic stress disorder [3]. On the other hand, with the overall aging of human society, cognitive impairment in the elderly, as represented by Alzheimer’s disease (AD), is evolving into a significant public health issue [4]. The most populous country in the world is China (about 18.32% of the world’s population), and it is rapidly transforming into an aging country. By 2050, there will be 400 million Chinese citizens aged 65+, 150 million of whom will be 80+ [5]. The prevention of cognitive decline in the Chinese elderly has also become an urgent medical issue. However, memory and cognitive loss problems in AD patients are usually treated with pharmacological approaches.

Nevertheless, many problems associated with taking these medicines have been described in these patients. The disease progresses and worsens even more, causing effects such as neuronal damage and mature neurons becoming immature [6,7,8]. Therefore, scholars have conducted a great deal of research in non-pharmacological treatment modalities that do not pose a risk to improving the ability to enhance learning and memory. One of the most direct and effective ways is to intervene in the daily diet.

Milk fat globule membrane (MFGM), a relatively new substance that encases milk fat globules, has a unique three-layer membrane structure and a thickness of about 10–50 nm [9]. In recent years, more and more scholars have focused their research on the biological activity of MFGM and its ability to enhance learning and memory. Previous studies indicated that diets supplemented with MFGM improved neurodevelopment and enhanced cognitive abilities in infants and newborn piglets, achieving similar effects to those in breastfed infants [10,11,12]. Similarly, an MFGM-supplemented formula has been reported by parents to be associated with lower behavioral problems in school-going children [13,14]. Additionally, O’Mahony et al. indicated that dietary intervention with MFGM significantly improved cognitive impairment caused by early stress in mice [15]. Furthermore, MFGM improved neurodevelopment in growth-restricted immature rats by upregulating the expression of hippocampal neurodevelopment-related genes (brain-derived neurotrophic factor (BDNF), dopamine receptor 1, (Drd1), glutamate receptor (GluR-1) and ST8 alpha-N-acetyl-neuraminide alpha-2,8-sialytransferase 4 (St8Sia4)) and improving T-Maze scores [16]. There is no doubt that the effects of MFGM on cognitive function are widely recognized. However, many previous studies are based on young animals or infants, and there is a lack of research on the adolescent stage. Moreover, the current studies lack a possible mechanism of memory and cognitive regulation based on proteomic upregulation and downregulation of proteins.

The hippocampus is the region of the brain that controls memory, learning, and cognition. Therefore, this study aimed to investigate the effects of MFGM intervention on behavioral, neuronal, and hippocampal proteomics in mice. The above can more completely elucidate the mechanisms by which MFGM improves mouse cognition and memory abilities.

## 2. Materials and Methods

### 2.1. Materials

MFGM was kindly donated by Hilmar Ingredients (Hilmar, CA, USA). All other chemicals and solvents were analytical grade unless otherwise stated.

### 2.2. Determination of Physicochemical Composition of MFGM

The determination methods of crude protein content, moisture, and primary amino acid composition and content of MFGM are based on the published literature [17]. The fat content was determined by the Soxhlet extraction method in accordance with the Chinese Standard GB 5009.6–2016. Furthermore, phospholipids, which are the major lipid components in MFGM, were measured for their main five glycolipids (phosphatidylcholine (PC), phosphatidylethanolamine (PE), phosphatidylinositol (PI), phosphatidylserine (PS), and sphingomyelin (SM)) contents according to the method described previously by Ferraris et al. [18].

### 2.3. Animals and Experimental Design

All animal husbandry and experimental designs were carried out in strict accordance with the Beijing Regulations on the Management of Laboratory Animals and approved by the Ethics Committee of Peking University School of Medicine (Approval No.: LA2021477). Male BALB/c mice (7–8 weeks old, SPF grade) were obtained from Beijing Weitong Lihua Experimental Technology Co., Ltd. (Beijing, China). The mice were fed standard chow and water ad libitum for one week in a temperature-controlled (23 ± 1 °C) and constant humidity (60 ± 10%) environment and subjected to a 12 h light–12 h dark cycle to acclimatize them to their new environment. Subsequently, the mice were randomly divided into two groups (*n* = 10) according to body weight (BW): (1) the control group (C); and (2) the MFGM group (M). The M group was given MFGM sterile saline solution (400 mg/kg BW) by intragastric gavage once a day. Meanwhile, the control group (Control) was administered equal volumes of sterile saline solution for 18 weeks successively with gavages. The body weight of each mouse was weighed and recorded every three days throughout the experiment. All efforts were made to minimize the number of animals used. All mice were used following protocols approved by the Institutional Animal Care and Utilization Committee.

### 2.4. Morris Water Maze (MWM) Experimental Design

The experiment followed the method described by Tucker et al. [19] with appropriate modifications. Briefly, a large circular tank filled with water (diameter = 120 cm, temperature = 25 °C) was prepared in advance, and the water surface was manually divided into four quadrants. Mice were placed at different random locations each time to train the ability to find a hidden/submerged escape platform (1 cm below the water level) in a circular tank using distal spatial cues. The monitoring time of mice swimming was set to 60 s. Mice that did not find the platform were guided to the platform and allowed to stay for 10 s. This procedure required four trials per day for four consecutive days with the platform fixed in the first quadrant. Afterwards, the underwater platform was removed from the tank, and the mice were retested and recorded. It is worth mentioning that, based on the fact that BALB/c is a white mouse, 20 g of food-grade melanin needs to be added to the water before the experiment so that the instrument can accurately record the mice swimming.

### 2.5. Histological Analysis of the Hippocampus

#### 2.5.1. Hematoxylin and Eosin (H&E) Staining

At the end of the MWM test, mice were euthanized by intraperitoneal injection of 50 mg/kg pentobarbital sodium [19]. Mice were stripped of their brains on ice immediately after losing consciousness, and the left hippocampus was fixed with 4% paraformaldehyde for subsequent staining analysis. The method of H&E staining was referred to in the previously published literature by Wang et al. and Geng et al. [20,21]. In brief, the fixed left hippocampus was trimmed, dehydrated in gradient alcohol, xylene transparency, and then embedded in paraffin. The thickness of the section was 5 µm, dried at 37 °C, and dewaxed in xylene. Morphological changes in mouse hippocampal cells were observed under a light microscope (Nikon Eclipse E100, Tokyo, Japan) and an imaging system (Nikon DS-U3, Tokyo, Japan).

#### 2.5.2. Nissl Staining 

Nissl staining was used to observe the number of neurons in the hippocampal region. Pre-section processing was carried out in the same way as H&E staining. The sections were deparaffinized three times consecutively (10 min each) in xylene. Subsequently, the slices were dehydrated with gradients of 100%, 95% and 80% ethanol, respectively, then, washed with distilled water three times in succession (5 min each). Finally, the sections were treated with Nissl staining solution for 5 min and then the above dehydration and washing steps were repeated and the sections were sealed with neutral balsam. Resin coverslips were used for section observation. The digital images were acquired with a microscope (Nikon Eclipse E100, Tokyo, Japan). Positive areas in the images were quantified, and the average optical density (AOD) was calculated using Image J 1.53k software (National Institutes of Health, Bethesda, MD, USA).

### 2.6. Proteomics Analyses in Hippocampal Tissue of Mice

#### 2.6.1. Tandem Mass Tag (TMT) Labeling

Three representative hippocampal tissues were selected from the two groups of mice. Protein sample preparation and TMT labeling were performed according to the method described by Wang et al. [21]. Briefly, hippocampal tissue was ground into a powder using liquid nitrogen and mixed well before being transferred to a centrifuge tube. Subsequently, the appropriate amount of lysate (including 8 M urea and 1% protease inhibitor mixture) was added to the centrifuge tube and sonicated three times on ice with a high-intensity processor. The supernatant was collected by centrifugation at 10,000× *g* for 30 min at 4 °C. Immediately after, the protein solution was reduced with 10 mM dithiothreitol (DTT) at 55 °C for 30 min, and incubated with 25 mM iodoacetamide (IAA) in the dark at room temperature for 60 min. The treated samples were digested using 1 M urea and trypsin (1:100 *w*/*w*) at 37 °C for 14 h. The digested peptides were vacuum-dried and dissolved in 50 μL triethylammonium bicarbonate buffer (TEAB; 200 mM, pH 8.5) for TMT labeling. Finally, the peptides were labeled with TMT reagent (0.8 mg powder consisting of TMT dissolved in 41 μL acetonitrile) and incubated at room temperature for 60 min. The reaction was then terminated by adding 5% hydroxylamine and incubating at room temperature for 15 min.

#### 2.6.2. Liquid Chromatography-Tandem Mass Spectrometry (LC-MS/MS) and Bioinformatics Analysis

The LC-MS/MS analysis was performed by referring to the literature previously published by Zhong et al. [22]. In detail, the enriched peptide was dissolved in 0.1% formic acid (FA) and loaded onto a fractionation C18 column (Wasters BEH, 4.6 × 250 mm, 5 μm) on a Rigol L3000 HPLC system. Then, the bound peptide was eluted into ten different fractions. All fractions were vacuum-dried and dissolved in 20 μL of 0.1% FA. Each fraction was loaded onto a C18 Nano-Trap column (75 μm × 2 cm, 3 μm) of an EASY-nLCTM 1200 UHPLC (Thermo-Fisher Scientific, San Jose, CA, USA) system. Peptides were eluted on a reversed-phase C18 column (150 μm × 15 cm, 1.9 μm) with a 90 min gradient of 6–100% buffer solution (80% acetonitrile and 0.1% formic acid) and 0.1% FA. Finally, the eluates were subjected to LC-MS/MS analysis. In addition, to characterize the properties of differential express proteins (DEPs), the Gene Ontology (GO) enrichment analysis (https://www.geneontology.org/ (accessed on 20 September 2022)) and Kyoto Encyclopedia of Genes and Genomes (KEGG) pathway enrichment analysis were performed. 

### 2.7. Statistical Analysis

Unless otherwise specified, all measurements were performed in triplicate. Data were analyzed by one-way analysis of variance, and the results were expressed as mean ± standard deviation. Differences between control and experimental groups were determined by using Student’s *t*-test. *p* < 0.05 was considered statistically significant. Statistical analysis was performed using SPSS software (24.0, SPSS Inc., Chicago, IL, USA). The images were drawn using the Origin software (Origin Lab Co., Pro.9.0, San Diego, CA, USA).

## 3. Results

### 3.1. Composition Analysis of MFGM

Crude protein, fat, phospholipid, moisture, and amino acid composition and content are the basic indicators for evaluating MFGM (Table 1). The crude protein, fat, and moisture contents of MFGM were 66.47%, 14.98%, and 5.79%, respectively. Meanwhile, the primary amino acids in MFGM are glutamate, proline, aspartate, lysine, leucine, valine, tyrosine, phenylalanine, isoleucine, and serine, accounting for 16.42%, 10.83%, 10.53%, 9.44%, 8.32%, 6.72%, 5.54%, 5.08%, 4.69% and 4.18% of the total protein, respectively. The composition of these components indicated that MFGM could meet the basic nutritional needs of mice during the experiment. It is noteworthy that the contents of PC, PE, PI, PS and SM are 1.97%, 2.61%, 0.14%, 0.88% and 1.86% respectively. The content of major phospholipids reached a high percentage of 7.46%. MFGM has been reported to have beneficial effects on brain development and function, possibly due to its high phospholipid content [10]. 

### 3.2. Growth and Morris Water Maze Performance

**Figure 1:**
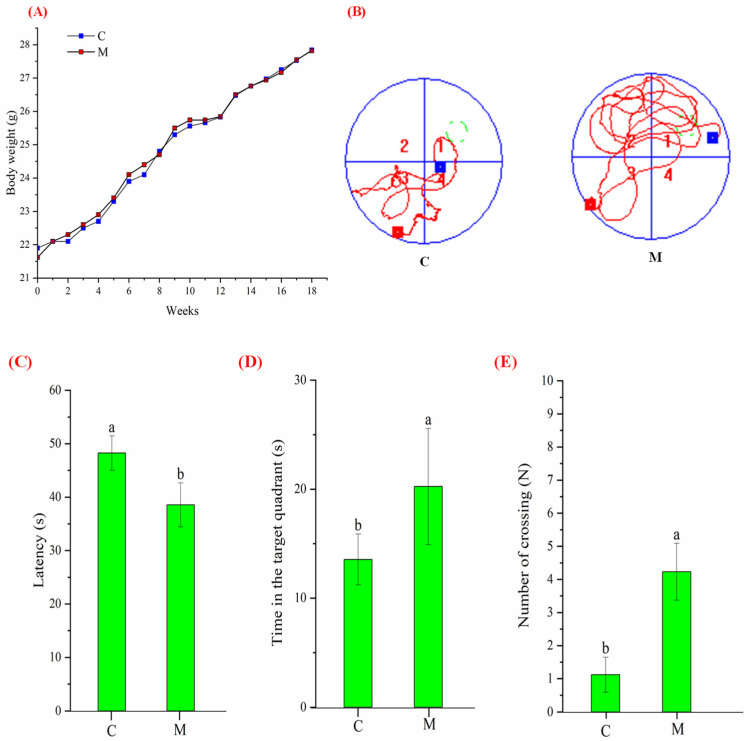
The effects of body weight, learning, and memory capacity after 18 weeks of MFGM intervention in mice: (**A**) changes in body weight of mice with feeding time; (**B**) schematic diagram of mice movement trajectory in the space exploration experiment. The red square represents the point where the mouse entered the water, the blue square represents the point where the mouse was located after the 60 s timing, and the green circle dotted line represents the original platform placement point; (**C**) analysis of the exploration time; (**D**) analysis of time in the target quadrant; and (**E**) analysis of the number of times crossing. All experiments were in triplicate. Different lowercase letters (a, b) in (**C**–**E**) means significant statistical differences. Capital letters “C” and “M” in (**A**,**B**) represent control and experimental groups, respectively. Data are presented as mean ± SEM, *p* < 0.05 compared to the control group.

To confirm whether MFGM poses a risk of obesity in mice, we monitored the changes in body weight throughout the 18-week study. As shown in Figure 1A, the body weight of the control and experimental groups increased with the rearing time, and there was no significant difference in growth rate and final body weight. On the other hand, to assess MFGM intervention’s effect on mice’s cognitive behavior, we performed the MWM test and analyzed the experimental trajectory and data. The trajectory of group M looks more disordered than groups C (Figure 1B). During the spatial exploration experiment, the M took less time (38.55 ± 4.13 s) to find the original underwater platform (Figure 1C), stayed longer (20.24 ± 5.34 s) in the target quadrant (Figure 1D) and crossed the original platform location more often (4.23 ± 0.86, Figure 1E). However, the corresponding data for the control mice were 48.25 ± 3.22 s, 13.55 ± 2.33 s, and 1.12 ± 0.53, respectively. Meanwhile, there was no apparent difference in body weight and no external signs of pathological injury or disease, indicating that the mice were healthy before the model was constructed. The above findings suggest that MFGM can enhance the learning and spatial memory abilities of BALB/c mice.

### 3.3. Effect of MFGM on Hippocampus Histological Analysis

**Figure 2:**
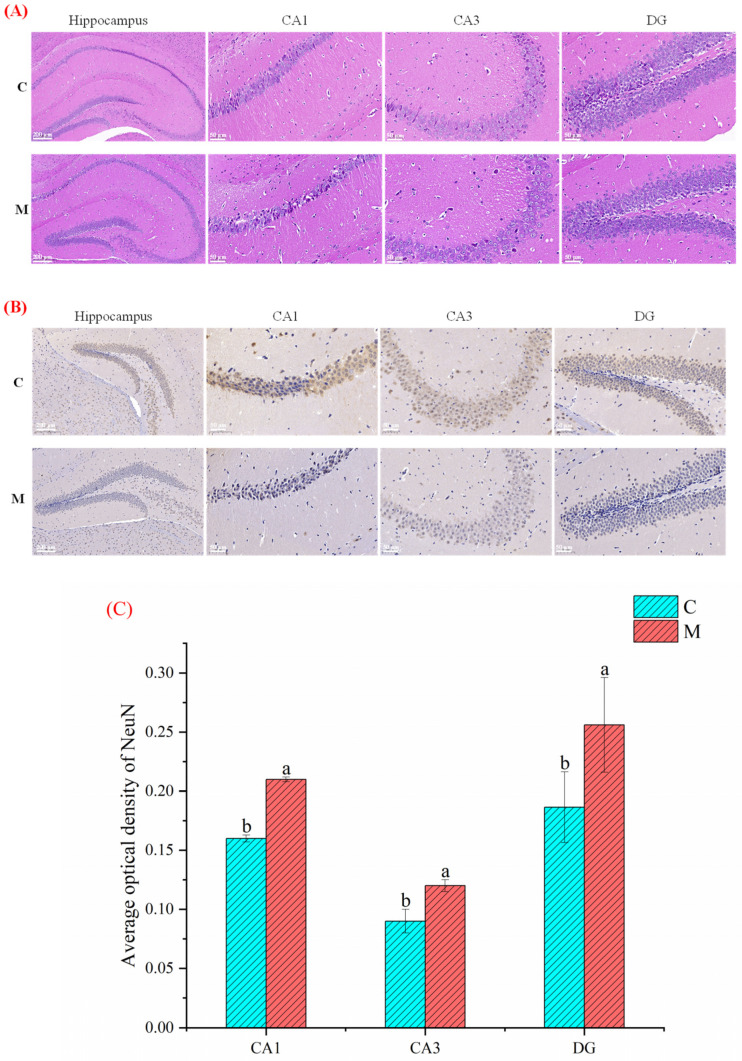
Effect of MFGM on hippocampal neurons in mice: (**A**) H and E; (**B**) Nissl staining of the mice’s hippocampus, dentate gyrus (DG), CA1 and CA3 region. Scale bars = 50 μm in CA1, CA3, and DG; Scale bars = 200 μm in hippocampus; and (**C**) The average optical density (AOD) of NeuN (neuronal nuclei) in the CA1, CA3, and DG region. All experiments were in triplicate. Different lowercase letters (a, b) in (**C**) means significant statistical differences. Capital letters “C” and “M” in (**A**,**B**) represent control and experimental groups, respectively. Data are presented as mean ± SEM, *p* < 0.05 compared to the control group.

To investigate the effect of MFGM intervention on neurons in the mice hippocampus, we observed the morphological changes of hippocampal neurons by H&E staining. As shown in Figure 2A, the number of neurons was significantly increased in group M compared with group C, especially in the hippocampus’s dentate gyrus (DG) area. Also, a small number of neurons in the hippocampus of group C had shrunk. Strikingly, it was found that neurons in the whole brain areas, including the DG, CA1, and CA3 regions of the hippocampus in group M were more intensive than in group C. On the other hand, the results of Nissl staining specified that the hippocampal neuronal cells in group M were more evenly distributed with regular morphology and significantly more Nissl bodies than in groups C (Figure 2B). At the same time, we quantified the average optical density (AOD) of neuronal nuclei (NeuN) using image J software (National Institutes of Health, Bethesda, MD, USA). The expression level of NeuN in group M was distinctly higher than in groups C (Figure 2C), indicating that group M had stronger spatial memory and cognitive abilities. 

### 3.4. Proteomic Analysis

#### 3.4.1. Full Protein Function Annotation

**Figure 3:**
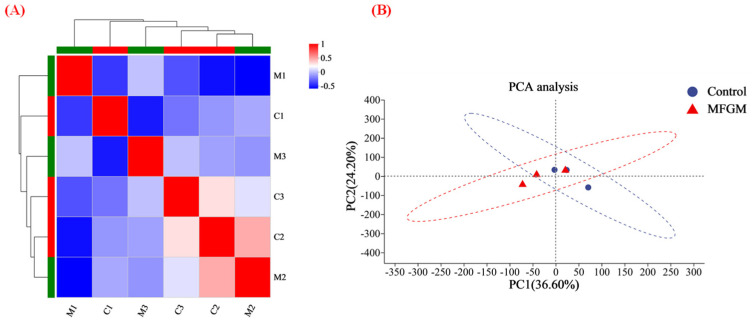
Functional annotation of whole proteins: (**A**) heat map of the correlation between M and C (C1, C2, C3 and M1, M2, M3 represent parallel samples of the control and experimental groups, respectively); and (**B**) PCA analysis. “MFGM” in (**B**) represent experimental groups.

TMT quantitative proteomics was used to investigate the proteomic differences between groups C and M. A total of 7067 proteins were identified in the two treatment groups. Pearson correlation analysis and PCA were used to study the similarity of the whole proteins in the two treatment groups based on the type and content of proteins in each treatment group. Figure 3A shows the highest similarity between the parallel samples, and the whole protein variability was higher in groups C and M. The PCA results in Figure 3B demonstrate that PC1 and PC2 can reflect 60.8% of the total information. The extraction was relatively complete, indicating that these two PCs can replace the original components to reflect the sample information. PC1 had the most significant impact on the overall results, representing 36.6% of all information. The above results indicated that MFGM significantly altered protein expression in the hippocampus of mice. 

#### 3.4.2. MFGM Altered the Hippocampus Proteomic Profiles

**Figure 4:**
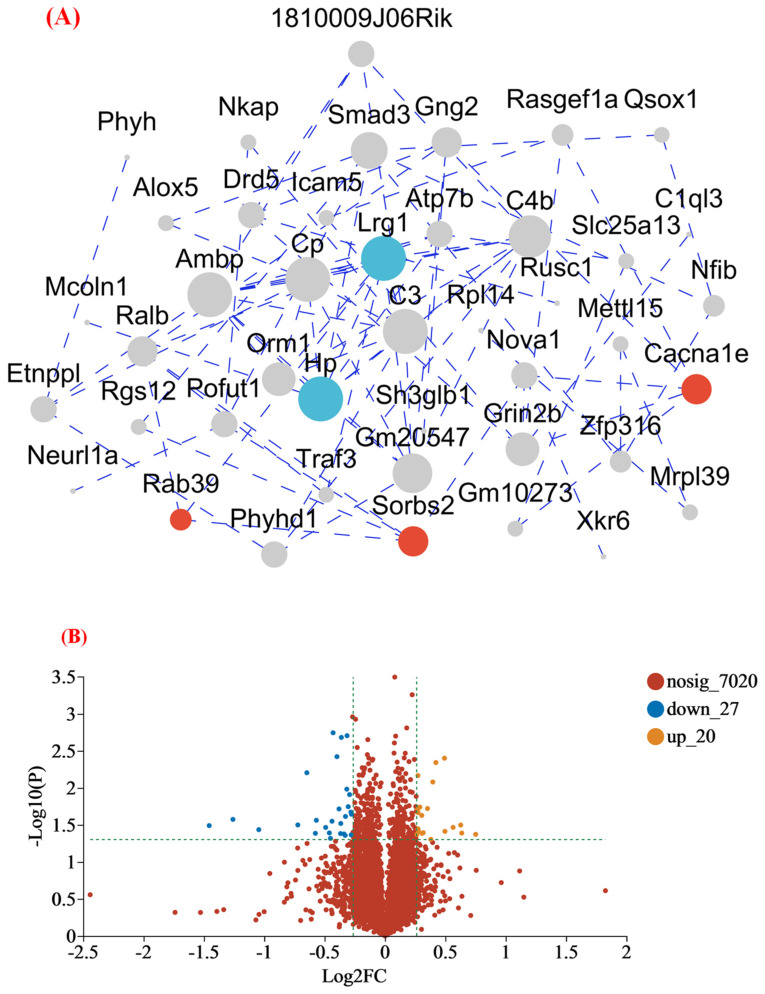

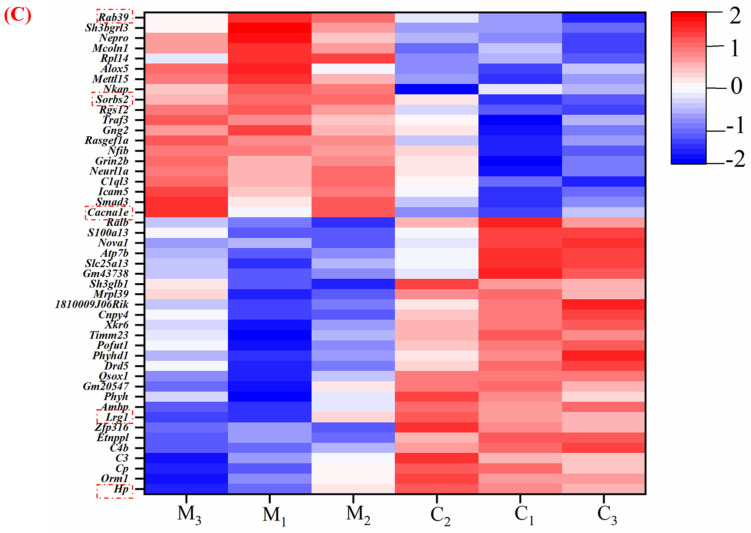
(**A**) Interaction map of differential expression proteins; (**B**) volcano plot of the differentially expressed protein in the M group compared to the C group; and (**C**) the differentially expressed protein expression heatmap. The blue represents the lower expression level. The white represents the median value, and the red refers to the higher expression level. (C1, C2, C3 and M1, M2, M3 represent parallel samples of the control and experimental groups, respectively). Genes in red dashed boxes in C are strongly correlated with memory and cognitive ability.

This study used proteomic techniques to determine how MFGM improves mouse cognition and spatial memory. At *p* < 0.05, proteins with >1.2 fold−change (FC) values were considered as significantly upregulated, while proteins with <0.83 FC values were considered as significantly downregulated [20]. The expression of 7067 proteins was identified from the protein database. The most upregulated proteins were Rab39 (1.55), Sorbs2 (1.55), and Cacna1e (1.48). Meanwhile, the most downregulated proteins were Hp (0.36), and Lrg1 (0.48). To better understand the protein−protein interactions between DEPs in the hippocampus, protein interaction analysis was performed using the string database to identify the protein interactions in the protein set (Figure 4A). According to a degree, the top two genes in the network were Hp and Lrg 1, and the proteins of these genes exhibited close interactions. Moreover, Figure 3B is a volcanic map of all the differentially expressed proteins, illustrating the close correlation between various proteins. Compared to group C, it also indicated that 20 proteins were upregulated and 27 were downregulated in group M (Figure 4B). At the same time, to visualize the expression of proteins in the differential protein set in each group of samples, we performed a clustering heat map analysis (Figure 4C). The blue represents the lower expression level. The white represents the median value, and the red refers to the higher expression level. The genes with high expression levels in groups C (Lrg 1, Hp) and M (Rab 39, Sorbs 2, and Cacna 1e) are listed. These are consistent with the results of the upregulated and-downregulated proteins analyzed above, further suggesting that MFGM can improve cognition and spatial memory in mice by upregulating and downregulating the expression of related proteins.

#### 3.4.3. Functional Annotation of Differentially Expressed Proteins

**Figure 5:**
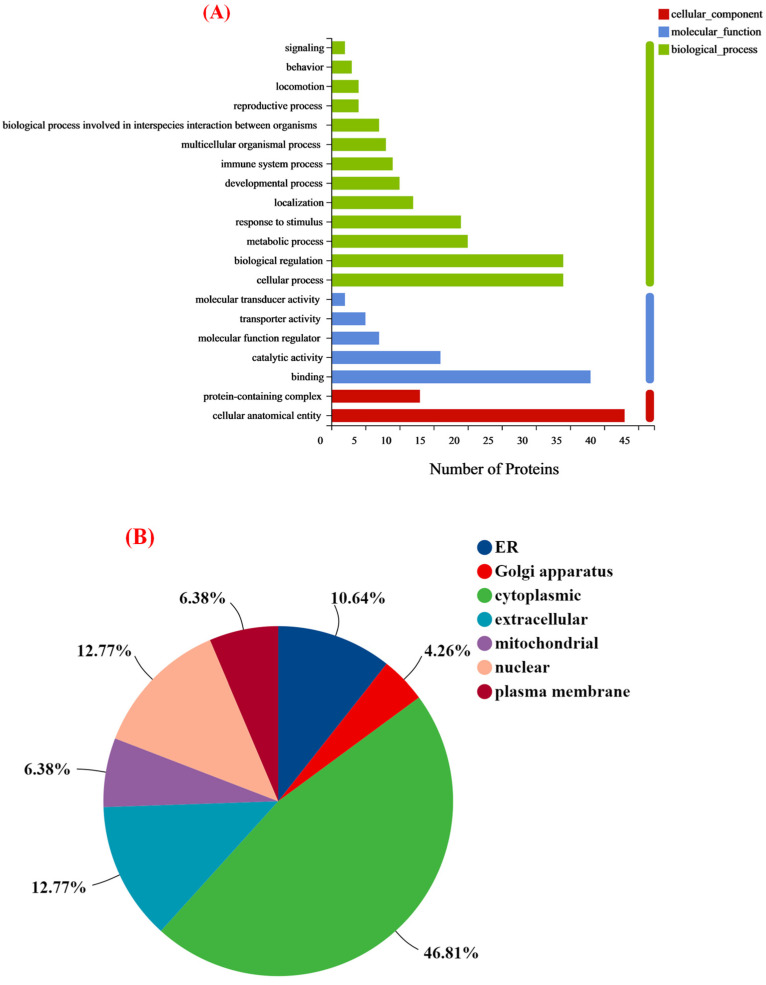

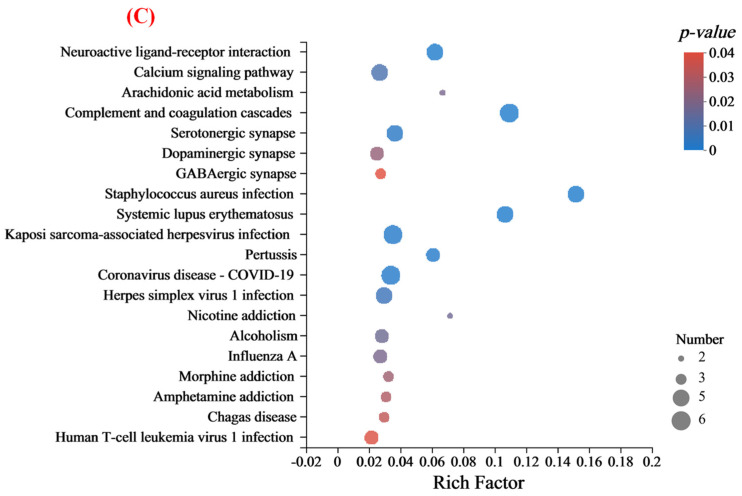
(**A**) Statistical distribution plot of differential expressed proteins in GO level 2 classification; (**B**) subcellular structural localization analysis of differential expressed proteins; and (**C**) KEGG bubble chart of differentially expressed proteins.

To better understand the functions and characteristics of the DEPs identified in this study, GO analysis was used to reveal the top GO terms. As shown in Figure 5A, the proteins with different expression proteins were classified into three sub-components: biological processes, molecular functions, and cellular components. In the biological process category, most DEPs were involved in the cellular process, biological regulation, metabolic process, and response to the stimulus. The cellular component category indicated that over half of the identified proteins were located in the cellular anatomical entity. For the molecular function part, many proteins identified were associated with binding and catalytic activity. Of course, the subcellular localization and classification of altered proteins indicated that the cytoplasmic, nuclear, and extracellular accounted for 70% of the above (Figure 5B). Furthermore, KEGG pathway analysis was performed to identify the associated signaling pathways. It mainly included neuroactive ligand-receptor interaction, calcium signaling pathway, complement and coagulation cascades, serotonergic synapse, and dopaminergic synapse (Figure 5C). Meanwhile, KEGG enrichment analysis of the DEPs identified a total of 202 pathways, with only 7 signaling pathways significantly enriched (*p* < 0.05), which might be associated with improved learning memory capacity in group M (Table 2). 

## 4. Discussion

The body weight of groups C and M increased with increasing experimental time, and there was no significant difference in growth rate and final weight. This result is similar to those of previous studies [23,24], indicating MFGM does not cause the risk of obesity in mice. On the other hand, the high phospholipids (~7.46%) in MFGM have been demonstrated to have a variety of health benefits, including improved cognitive abilities across the lifespan [25]. In our research, the motion trajectory of MWM in group M was more disordered compared with group C. Mice with a greater spatial memory for information learned during training will spend more time in areas proximal to the former location of the platform and may pass through that exact location (the “annulus”) multiple times [19]. During the spatial exploration experiment, the M took less time to find the original underwater platform, stayed longer in the target quadrant, and crossed the original platform location more often. These behavioral aspects of mice suggest that MFGM significantly improves their cognitive and spatial memory abilities. Previous studies have indicated that animals’ performance in MWM tests highly depends on hippocampal function [26]. The learning and memory abilities of animals are also inextricably linked to their hippocampus [16,24,27,28]. The hippocampus is closely associated with cognitive function, and the hippocampus’s DG, CA1, and CA3 regions are the main areas of neurogenesis in the brain [29]. Whether H and E staining or Nissl staining were performed, there were significantly more neurons (especially in the DG region) in group M, and the AOD value of NeuN was higher. This indicates that MFGM can slow down the apoptosis of neurons. These results suggest that MFGM may play a neuroprotective role by reducing neuronal apoptosis to enhance cognition and spatial memory in BALB/c mice [16]. 

Pearson correlation analysis and PCA were employed to analyze the similarity of whole proteins in groups C and M according to the types and contents of proteins in the two groups. The closer the Pearson correlation was to 1, the higher the similarity of protein composition between groups C and M [30]. In addition, 47 differentially expressed proteins (DEPs), including 20 upregulated and 27 downregulated proteins were identified. It cannot be ignored that phospholipids not only have a positive effect on behavior, but also participate in the upregulation of genes such as neurodevelopment in the hippocampus [31]. The most upregulated proteins were associated with memory and cognition [32,33,34,35]. Rab proteins are a vesicle transporter regulatory group. They are located in different membrane structures and regulate vesicle transport, affecting neuromodulation and cognitive ability by affecting the formation of synapses [36]. Deletion of Rab39b resulted in impairments of synaptic structures and functions, with reductions in NMDA receptors in the postsynaptic density [32]. NMDA receptors, ionotropic glutamate receptor, is central to memory consolidation and spatial processing in the hippocampus [37]. Thus, upregulated expression of Rab 39 may promote synaptic structure formation and increased function to improve memory and cognitive performance in mice. Furthermore, in a previous study, the deletion of Sorbs2 in mice reduced dendritic complexity and excitatory synaptic transmission in DG granule cells and impaired acoustic startle response and long-term memory. The Cacna1e gene encodes CaV2.3 channels, which are widely expressed throughout the nervous system and are located in presynaptic terminals, dendritic spines, and some extrasynaptic sites. Disruption of CaV2.3 channel function leads to increased anxiety-like behavior and impaired spatial memory [38]. On the other hand, the top two proteins in the interaction network according to the degree of protein interaction were Hp and lrg1 (Figure 4A), the two most downregulated proteins. Multiple studies have shown that the expression of these two proteins leads to higher cognitive impairment [39,40]. The findings of Guerrero-Berroa et al. suggest that patients with type 2 diabetes and poor glycemic control carrying the Hp 1-1 genotype may be at increased risk of cognitive impairment, particularly in the attention/working memory domain [39]. Also, compelling evidence indicates that Lrg1 can promote apoptosis and autophagy to exacerbate ischemia/reperfusion-induced brain injury through the transforming growth factor beta (TGFβ)/SMAD signaling pathway [41]. In another study, Akiba et al. indicated that Lrg overexpression in the hippocampus can lead to synaptic dysfunction and to memory impairment with age [42].

**Figure 6:**
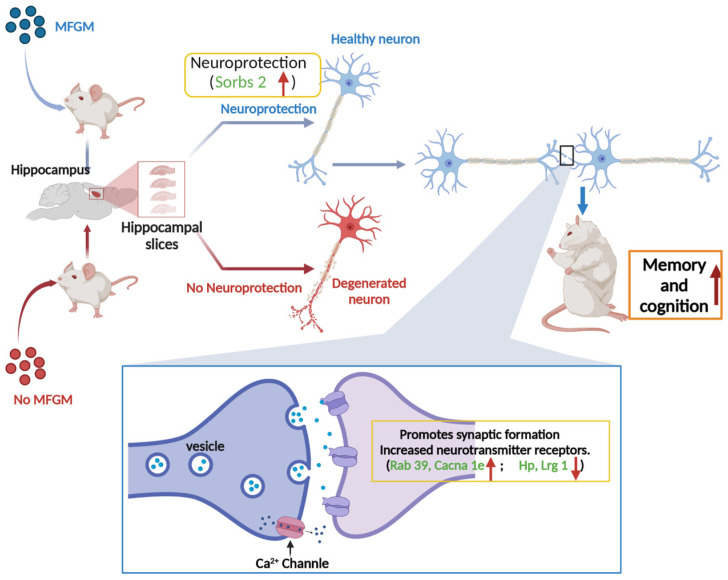
Schematic diagram of synaptogenesis and neurotransmission facilitation in cognitive and memory function enhancement by MFGM intervention.

KEGG pathway analysis was performed to identify the associated signaling pathways to understand better the functions and characteristics of the DEPs identified in this study. It has been shown that the disruption of genes involved in “neuroactive ligand-receptor interactions” leads to decreased memory function [43]. The results of Qiu et al. revealed a close link between behavioral changes and altered neurotransmitter receptor gene expression in Benzo(a)pyrene-treated rats [44]. Also, the calcium pathway regulates memory formation in neurons [45]. As observed in Table 2, “neuroactive ligand-receptor interactions” are one of the signaling pathways involved. It is positively correlated with memory and cognitive performance [46]. The possible mechanism of MFGM intervention to enhance mice’s cognitive and memory abilities is shown in Figure 6. In detail, the upregulation of Sorbs 2 may have a certain protective effect on neuronal cells, thereby delaying the natural apoptotic cycle of neuronal cells. Also, the upregulation of Rab 39 and Cacna 1e promotes the formation of synaptic structures, the increase of neurotransmitter receptors, and the smoothness of calcium channels. The downregulation of Hp and Lrg 1, two genes that inhibit synaptic conduction and neuromodulation, also promotes memory and cognition from another perspective.

Based on the above analysis, MFGM may mainly upregulate positively correlated proteins and downregulate proteins negatively correlated with cognition. On the other hand, it enhances the “neuroactive ligand-receptor interaction” pathway, thereby improving spatial memory and cognitive impairment. 

## 5. Conclusions

The present study showed that dietary supplementation by MFGM significantly improved memory and cognitive impairment in BALB/c mice. Mice supplemented with MFGM showed stronger spatial memory in the Morris water maze experiment compared to the control group. Furthermore, indications of H and E and Nissl staining suggest that MFGM reduces the loss of neuronal cell numbers. MFGM intervention may enhance learning and spatial memory abilities in mice by promoting synaptogenesis and neurotransmission. Our study provides novel insights and references to the potential mechanisms by which MFGM enhances learning and spatial memory abilities.

## Figures and Tables

**Table 1:** Basic composition analysis.

Composition	Content
Crude protein	66.47 ± 6.56%
Fat	14.98 ± 0.20%
Moisture	5.79 ± 0.14%
Glutamate	16.42 ± 0.01%
Proline	10.83 ± 0.01%
Aspartate	10.53 ± 0.04%
Lysine	9.44 ± 0.02%
Leucine	8.32 ± 0.02%
Valine	6.72 ± 0.01%
Tyrosine	5.54 ± 0.03%
Phenylalanine	5.08 ± 0.01%
Isoleucine	4.69 ± 0.03%
Serine	4.18 ± 0.04%
Threonine	3.78 ± 0.02%
Histidine	3.40 ± 0.03%
Arginine	3.14 ± 0.01%
Alanine	2.92 ± 0.01%
Methionine	2.59 ± 0.02%
Glycine	2.27 ± 0.02%
Cysteine	0.14 ± 0.01%
Phosphatidylcholine (PC)	1.97 ± 0.02%
Phosphatidylethanolamine (PE)	2.61 ± 0.01%
Phosphatidylinositol (PI)	0.14 ± 0.01%
Phosphatidylserine (PS)	0.88 ± 0.03%
Sphingomyelin (SM)	1.86 ± 0.02%

Data are shown as means ± standard deviation (*n* = 3).

**Table 2:** Results of KEGG enrichment analysis of differentially expressed proteins in hippocampus.

KEGG Description	DEPs Number	Pathway ID	*p* Value
Staphylococcus aureus infection	5	mmu05150	<0.01
Complement and coagulation cascades	6	mmu04610	<0.01
Systemic lupus erythematosus	5	mmu05322	<0.01
Neuroactive ligand-receptor interaction	5	mmu04080	<0.01
Pertussis	4	mmu05133	0.03
Coronavirus disease-COVID-19	6	mmu05171	0.03
Kaposi sarcoma-associated herpesvirus infection	6	mmu05167	0.04

## Data Availability

The data that support the findings of this study are available from the corresponding author upon reasonable request.
